# The regulatory role of CASZ1 in keratinocyte differentiation and skin barrier function in atopic dermatitis

**DOI:** 10.1016/j.gendis.2025.101767

**Published:** 2025-07-11

**Authors:** Xuemei Li, Yuqiong Huang, Changdeok Kim, Li Fu, Hongxiang Chen, Kyungeun Jung

**Affiliations:** a Department of Dermatology, Huazhong University of Science and Technology Union Shenzhen Hospital, Shenzhen, Guangdong 518052, China; b Guangdong Key Laboratory for Biomedical Measurements and Ultrasound Imaging, National-Regional Key Technology Engineering Laboratory for Medical Ultrasound, School of Biomedical Engineering, Shenzhen University Medical School, Shenzhen, Guangdong 518060, China; c Department of Dermatology, Shenzhen Second People’s Hospital, The First Affiliated Hospital of Shenzhen University Health Science Center, Shenzhen, Guangdong 518035, China; d Department of Dermatology, Chungnam National University School of Medicine, Chungnam National University Hospital, Daejeon 35015, South Korea; e Department of Dermatology, Zhongnan Hospital of Wuhan University, Wuhan University, Wuhan, Hubei 430071, China; f Department of Dermatology, Union Hospital, Tongji Medical College, Huazhong University of Science and Technology, Wuhan, Hubei 430022, China; g Guangdong Province Key Laboratory of Regional Immunity and Diseases, Department of Pharmacology and Shenzhen University International Cancer Center, Shenzhen University Medical School, Shenzhen, Guangdong 518060, China

The skin, being the largest organ of the human body, serves a critical barrier function by preventing the intrusion of harmful external substances and minimizing moisture loss. The epidermis consists of a stratified epithelium of continuously differentiating keratinocytes (KCs), which originate from the basal layer and progressively migrate upwards to form the stratum corneum. Proper epidermal differentiation is essential for maintaining the skin’s barrier function.^1^ However, disruptions in this process can lead to various skin diseases, including psoriasis, atopic dermatitis (AD), and squamous cell carcinoma.^2^ Thus, elucidating the regulatory network that governs epidermal differentiation is crucial for understanding the pathogenesis of these skin diseases.

Recent studies have shown that multiple transcription factors play critical roles in KC differentiation, including IRF6, GRHL3, NOTCH1, MAF/MAFB, PRDM1, OVOL1, ZNF750, KLF3, and KLF4.^3^ However, the complete regulatory mechanism has yet to be fully elucidated. This study aims to explore the role of the transcription factor CASZ1 (Castor zinc finger 1) in KC differentiation and skin barrier function and further understand its potential mechanisms in skin diseases.

To identify regulators of epidermal differentiation, we first compared the RNA expression differences in primary human KCs between undifferentiated and differentiated states using two datasets from the GEO database. In both datasets, we found that the expression of CASZ1 significantly increased following KC differentiation (Fig. 1A, B). Additionally, at the mRNA level, CASZ1 was significantly positively correlated with the late differentiation markers filaggrin (FLG) and involucrin (IVL) (Fig. 1C, D). To validate these findings, we conducted *in vitro* KC differentiation induction experiments (Fig. 1E). Using reverse transcription PCR and western blotting techniques, we analyzed the expression of CASZ1 and the late differentiation markers IVL and FLG during differentiation. The results showed that as KC differentiation progressed, the expression of CASZ1 significantly increased, along with notable increases in the expression of IVL and FLG (Fig. 1F–H). The mRNA and protein expression levels were quantified (Fig. 1G–I). These findings suggest that CASZ1 may play an important role in the late stages of KC differentiation.

**Figure 1:**
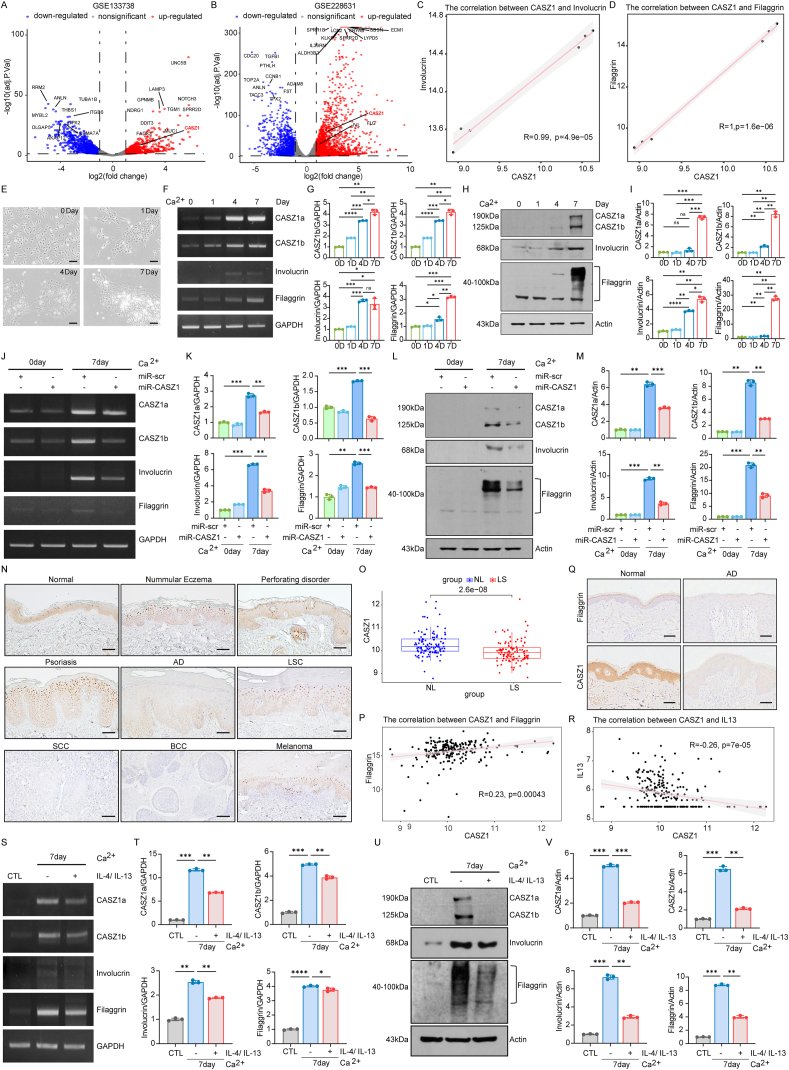
CASZ1 in keratinocyte differentiation and skin barrier function: Implications for skin diseases and inflammatory regulation. **(A, B)** Volcano plots of GSE133738 and GSE228631. Genes with an adjusted *P*-value (adj.P.Val) < 0.05 and a log_2_ fold change (logFC) > 1 were considered significantly differentially expressed. Gray dots represent genes with no significant differential expression, blue dots indicate down-regulated genes, and red dots indicate up-regulated genes. **(C, D)** Pearson correlation scatter plots of CASZ1 with IVL and FLG in GSE228631. Immortalized human keratinocytes were treated with 1.2 mM calcium for 1, 4, and 7 days. **(E)** Morphology of KC differentiation. Scale bar = 100 μm. **(F)** The mRNA levels of CASZ1, IVL, and FLG were determined by reverse transcription PCR. **(G)** The expression of mRNA was quantified and normalized to GAPDH, and the data were expressed as a fold change relative to a control (mean ± SEM; *n* = 3). **(H)** The protein levels of CASZ1, IVL, and FLG were analyzed by western blotting. **(I)** The expression of protein was quantified and normalized to actin, and the data were expressed as a fold change relative to a control (mean ± SEM; *n* = 3). Immortalized human keratinocytes were transduced with the recombinant adenovirus expressing microRNA targeting CASZ1 (miR-CASZ1). **(J)** The mRNA levels of CASZ1, IVL, and FLG were determined by reverse transcription PCR. **(K)** The expression of mRNA was quantified and normalized to GAPDH, and the data were expressed as a fold change relative to a control (mean ± SEM; *n* = 3). **(L)** The protein levels of CASZ1, IVL, and FLG were analyzed by western blotting. **(M)** The expression of protein was quantified and normalized to actin, and the data were expressed as a fold change relative to a control (mean ± SEM; *n* = 3). **(N)** Representative immunohistochemical images of CASZ1 in skin samples from normal and various skin diseases. Scale bar = 100 μm. **(O)** Box plot of CASZ1 expression in non-lesional and lesional samples in GSE193309. **(P)** Pearson correlation scatter plots of CASZ1 with FLG in GSE193309. **(Q)** Representative immunohistochemical images of CASZ1 in skin samples from normal and atopic dermatitis. Scale bar = 100 μm. **(R)** Pearson correlation scatter plots of CASZ1 with IL13 in GSE193309. Immortalized human keratinocytes were induced to differentiate for seven days, with simultaneous treatment of 50 ng/mL IL-4 and IL-13 for the same duration. **(S)** The mRNA levels of CASZ1, IVL, and FLG were determined by reverse transcription PCR. **(T)** The expression of mRNA was quantified and normalized to GAPDH, and the data were expressed as a fold change relative to a control (mean ± SEM; *n* = 3). **(U)** The protein levels of CASZ1, IVL, and FLG were analyzed by western blotting. **(V)** The expression of protein was quantified and normalized to actin, and the data were expressed as a fold change relative to a control (mean ± SEM; *n* = 3). ∗∗∗∗*P* < 0.0001, ∗∗∗*P* < 0.001, ∗∗*P* < 0.01, and ∗*P* < 0.05; ns, not significant. CASZ1, Castor zinc finger 1; FLG, filaggrin; IVL, involucrin; SEM, standard error of the mean; IL-4/13, interleukin 4/13.

CASZ1, also known as ZNF693, is a C2H2-type zinc finger transcription factor known to play significant roles in the development and disease of neural and cardiovascular tissues.^4^ Recent studies using short hairpin RNA (shRNA) to silence CASZ1 expression conducted a six-day KC differentiation experiment, followed by RNA sequencing and differential gene expression analysis. The results showed that among the 997 down-regulated differentially expressed genes under CASZ1 silencing conditions, 72% overlapped with differentially expressed genes up-regulated during KC differentiation. Gene Ontology (GO) analysis further revealed that these genes, down-regulated due to CASZ1 silencing, were significantly enriched in epidermal differentiation-related GO terms, such as KC differentiation, keratinization, and programmed cell death.^3^ These data indicate that CASZ1 is essential for the expression of late epidermal differentiation genes.

To further elucidate the function of CASZ1 in KC differentiation, we employed miRNA technology to knock down CASZ1 expression and induced KC differentiation for seven days. The knockdown efficiency of CASZ1 was assessed in three independent experiments. The average knockdown rates of CASZ1a and CASZ1b were 39.2% ± 3.8% and 65.1% ± 3.5%, respectively, at the mRNA level (Fig. 1J), and 44.0% ± 0.6% and 65.1% ± 1.8%, respectively, at the protein level (Fig. 1L), indicating consistent and effective miRNA transduction. We then analyzed its impact on the expression of KC differentiation markers IVL and FLG. The results showed that CASZ1 knockdown significantly inhibited the mRNA and protein expression of IVL and FLG (Fig. 1J–L). The mRNA and protein expression levels were quantified (Fig. 1K–M). These findings indicate that CASZ1 plays a promotive role in KC differentiation, particularly in the late stages of the differentiation process.

The study demonstrated that by constructing a genome-wide binding map, CASZ1 mainly bound to gene bodies and promoter regions. CASZ1-dependent genes include key transcription factors and keratinization genes in epidermal differentiation, such as KLF3, PRDM1, LCE3D, and FLG, which play important roles in epidermal differentiation and the establishment of barrier function.^3^

To investigate the role of CASZ1 in skin barrier-related diseases, we performed immunohistochemical staining analysis on tissue samples from patients with various skin barrier dysfunctions. In our experiments, we observed that CASZ1 was highly expressed in the epidermis of normal skin, with lower expression in the nuclei of basal layer KCs. Its expression gradually increased towards the stratum corneum, which is consistent with our previous cell-based findings suggesting that CASZ1 plays a role in regulating terminal differentiation. The expression of CASZ1 in nummular eczema, perforation disorder, psoriasis, and lichen simplex chronicus skin was similar to that in normal skin, showing a gradual increase from the basal layer to the stratum corneum. However, no expression of CASZ1 was detected in poorly differentiated skin tumors, such as squamous cell carcinoma, basal cell carcinoma, and melanoma. Notably, in the epidermis of patients with AD, a disease of long-standing interest to our research group, CASZ1 expression was markedly reduced (Fig. 1N). Furthermore, we analyzed the non-lesional and lesional data of AD patients from the GEO database. We found that CASZ1 was significantly down-regulated in the lesional skin of AD patients (Fig. 1O). Additionally, in the skin of AD patients, CASZ1 was positively correlated with FLG (Fig. 1P). To further investigate the relationship between CASZ1 and FLG in AD, we performed immunohistochemical analysis on skin lesions from AD patients with FLG mutations. Our results showed that in these patients, CASZ1 expression was significantly reduced, with no detectable expression in the epidermis of the affected skin areas (Fig. 1Q). This observation suggests a potential link between the loss of FLG and the down-regulation of CASZ1 in AD, and CASZ1 may play an important role in the pathogenesis of skin barrier dysfunction in AD.

In AD, the inflammatory factors IL-4 and IL-13 promote inflammation in the skin while inhibiting the differentiation process of KCs. This inhibition results in decreased expression of key proteins essential for skin barrier formation, such as FLG and IVL, thereby weakening the skin’s structural integrity and barrier function.^5^ To investigate whether the key inflammatory factors IL-4 and IL-13 in AD affected CASZ1 expression, we first analyzed the aforementioned AD dataset and found that CASZ1 expression was significantly negatively correlated with IL-13 in AD (Fig. 1R). To further verify this result, we treated KCs with IL-4 and IL-13, and the results showed that these cytokines significantly inhibited the mRNA and protein expression of CASZ1, IVL, and FLG (Fig. 1S–U). The mRNA and protein expression levels were quantified (Fig. 1T–V). This suggests that in AD, inflammatory factors IL-4 and IL-13 may inhibit CASZ1 expression, thereby disrupting the KC differentiation process and leading to impaired skin barrier function.

In summary, this study demonstrates that CASZ1 expression significantly increases during KC differentiation and plays a critical regulatory role in the late differentiation stage. We found that CASZ1 expression was significantly reduced in the epidermal tissues of AD patients, especially in those with FLG mutations, further suggesting the potential role of CASZ1 in skin barrier dysfunction diseases. Finally, our study also showed that in AD, inflammatory factors, such as IL-4 and IL-13, inhibited the normal differentiation process of KCs by suppressing CASZ1 expression. In conclusion, the key regulatory role of CASZ1 in KC differentiation and skin barrier function provides a new perspective for research. Future studies should further explore the specific mechanisms of CASZ1 in skin diseases and its potential as a therapeutic target. These studies will not only help reveal the pathogenesis of skin diseases but may also provide new ideas and methods for clinical treatment.

## CRediT authorship contribution statement

**Xuemei Li:** Writing – review & editing, Visualization, Supervision, Resources, Methodology, Funding acquisition, Data curation, Writing – original draft, Validation, Software, Project administration, Investigation, Formal analysis, Conceptualization. **Yuqiong Huang:** Visualization, Methodology, Formal analysis, Writing – original draft, Validation, Investigation, Data curation. **Changdeok Kim:** Project administration, Conceptualization, Writing – review & editing, Data curation. **Li Fu:** Data curation, Writing – review & editing. **Hongxiang Chen:** Writing – review & editing, Visualization, Software, Project administration, Investigation, Formal analysis, Conceptualization, Writing – original draft, Supervision, Resources, Methodology, Funding acquisition, Data curation. **Kyungeun Jung:** Writing – review & editing, Visualization, Software, Project administration, Investigation, Formal analysis, Writing – original draft, Supervision, Resources, Methodology, Funding acquisition, Data curation, Conceptualization.

## Ethics declaration

All participants were enrolled from the Chungnam National University Hospital, and this study design was approved by the Institutional Review Board of Chungnam National University Hospital (IRB 2016–07-009).

## Data availability

The authors confirm that the data supporting the findings of this study are available within the article and its supplementary materials.

## Funding

This study was supported by the 10.13039/100014717National Natural Science Foundation of China (No. 82173423), National Science Foundation Projects of Guangdong Province, China (No. 2024A1515012346), Shenzhen Basic Research Project (Natural Science Foundation) (Guangdong, China) (No. JCYJ20220530141802005, JCYJ20230807115912025), Shenzhen Nanshan District Science and Technology Major Project (Excellent Youth Fund) (Guangdong, China) (No. NSZD2023030), and Scientific Research Project of Huazhong University of Science and Technology Union Shenzhen Hospital (Guangdong, China)) (No. YN2021001).

## Conflict of interests

The authors declared no conflict of interests.
