# Supplementary material for: The regulatory role of CASZ1 in keratinocyte differentiation and skin barrier function in atopic dermatitis
Source: Genes Dis. 2025 Jul 11;13(1):101767. doi: 10.1016/j.gendis.2025.101767 (PMC12466131; doi:10.1016/j.gendis.2025.101767)
Supplement: Multimedia component 1 [file mmc1.docx]

**Supplementary Material and Methods**

**GEO data analyses**

The data used in this study were obtained from the Gene Expression Omnibus (GEO) database (accessible at: [https://www.ncbi.nlm.nih.gov/geo/](https://www.ncbi.nlm.nih.gov/geo/)). GSE133738 contains transcriptome sequencing results of two undifferentiated and two 48-hour differentiated neonatal primary human keratinocytes; GSE228631 provides RNA sequencing (RNAseq) data of primary neonatal human keratinocytes under non-differentiated or differentiated conditions, from which we extracted transcriptome sequencing results of three undifferentiated keratinocytes and three 92-hour differentiated keratinocytes. GSE193309 includes transcriptome sequencing results of non-lesional and lesional skin from AD patients and healthy controls, from which we extracted 116 non-lesional and 111 lesional transcriptome sequencing results. We used the DESeq2 package in R for normalization and differentially expressed genes (DEGs) analysis, with the statistical significance threshold set at |log2FC| > 1 and p-value < 0.05. Subsequently, volcano plots of expression data were constructed using the ggplot and ggrepel packages; box plots of specific gene expressions were created using the ggplot and ggpubr packages; Pearson correlation coefficients and significance were analyzed using the cor() function and ggpubr package, and correlation scatter plots were constructed.

**Cell culture and immortalisation**

Human skin tissues were obtained under the written informed consent of the donors. All procedures were approved by the Institutional Review Board (IRB) of Chungnam National University Hospital (IRB No.1011-135). Primary keratinocytes were isolated from the epidermis and then immortalized using a recombinant retrovirus expressing simian virus 40 T antigen. Immortalized human epidermal keratinocytes were routinely cultured in keratinocyte serum free medium supplemented with bovine pituitary extract and recombinant human epidermal growth factor (Life Technologies Corporation, Grand Island, NY, USA).

**Reverse transcription-polymerase chain reaction (RT-PCR)**

Total RNA was isolated from sebocytes using the Easy-blue RNA extraction kit (Intron Biotechnology, Seongnam, Korea). Two μg of total RNA was reverse transcribed with Moloney-murine leukemia virus (M-MLV) reverse transcriptase (RTase) (Elpis Biotech, Daejeon, Korea). A portion of the cDNA was taken and used in a PCR reaction. The sequences of the primers are as follows:

CASZ1a: 5’-CACGCATCACCAAGAGAGAA-3’ and 5’-CTGAGGTTGGAGGCTCAGTC -3’;

CASZ1b: 5’-AAGCCGCACACATGACAATA -3’ and 5’-GATGCTGCTGCTCCTACCTC-3’; Involucrin: 5’-TGCTCTGGGTTTTCTGCTTT-3’ and 5’-GATGTCCCAGCAACACACAC-3’; Filaggrin: 5’-TTGCCATCAGATGACCTTGA-3’ and 5’-GGACACAAGCACAGAAAGCA-3’; GAPDH: 5’-GTCAGTGGTGGACCTGACCT and 5'-AGGGGTCTACATGGCAACTG-3’. **The intensity of mRNA bands was quantified using Image J software. The expression levels of the target mRNA were normalized to the loading control GAPDH to account for variations in mRNA loading. The normalized expression levels were calculated as the ratio of the target protein band intensity to the GAPDH intensity. Each experiment was independently repeated three times, and the data are presented as mean ± standard deviation.**

**Western blot Analysis** **and Quantification**

Cells were harvested by centrifugation and then lysed in the Pro-prep protein extraction solution (Intron Biotechnology). After vigorous pipetting, the extract was centrifuged at 15,000 rpm for 15 minutes. Total protein was measured using the BCA protein assay kit (Thermo Fisher Scientific). Samples were subjected to sodium dodecyl sulfate-polyacrylamide gel electrophoresis (SDS-PAGE), transferred to nitrocellulose membrane, and incubated with the appropriate antibody at 4°C overnight with gentle agitation. The blot was then incubated with peroxidase-conjugated secondary antibody for 1 hour at room temperature and visualized by enhanced chemiluminescence (Intron Biotechnology). The primary antibodies used in this study were: CASZ1, Filaggrin, β-actin (Santa Cruz Biotechnologies, Santa Cruz, CA, USA); Involucrin (Invitrogen, Life Technologies, CA, USA). **The intensity of protein bands was quantified using Image J software. The expression levels of the target proteins were normalized to the loading control Actin to account for variations in protein loading. The normalized expression levels were calculated as the ratio of the target protein band intensity to the Actin intensity. Each experiment was independently repeated three times, and the data are presented as mean ± standard deviation.**

**Skin Sample Selection**

**To explore the role of CASZ1 in skin barrier-related diseases, tissue paraffin blocks from patients diagnosed with various skin conditions, including nummular eczema, perforation disorder, psoriasis, and lichen simplex chronicus, were obtained from the pathology department. The diagnosis was based on a combination of histopathological examination and clinical presentation, following established diagnostic criteria for each disease. Only well-documented cases were selected for analysis. Control samples were selected from healthy skin adjacent to excised lesions or non-diseased individuals matched for age and gender. Ethical approval for the use of these tissue samples was obtained from the Chungnam National University Hospital, and this study design was approved by the Institutional Review Board of Chungnam National University Hospital (IRB 2016–07-009).**

**Immunohistochemistry**

Tissue samples were fixed with 10% formaldehyde, embedded in paraffin, and cut into 4 μm thick sections. Sections were deparaffinized in xylene and then rehydrated using an alcohol series. For IHC, sections were fifirst treated with 3% H2O2 to block the endogenous peroxidase and then incubated with IHC blocking solution (DAKO, Carpinteria, CA, USA). The sections were then reacted with CASZ1, Filaggrin antibody (Santa Cruz Biotechnologies, Santa Cruz, CA, USA) at 4°C for overnight, followed by horseradish peroxidase-conjugated secondary antibodies (DAKO). After washing, the sections were incubated with diaminobenzidine tetrachloride solution and counterstained with Mayer’s hematoxylin.

**Statistical analysis**

Data were evaluated statistically by one-way ANOVA using the software GraphPad Prism 9.5.0. Statistical significance was set at P<0.05. A representative experiment such as micrographs has been repeated at least two to three times.
